# Comparison between Highly Complex Location Models and GAMLSS

**DOI:** 10.3390/e23040469

**Published:** 2021-04-16

**Authors:** Thiago G. Ramires, Luiz R. Nakamura, Ana J. Righetto, Renan J. Carvalho, Lucas A. Vieira, Carlos A. B. Pereira

**Affiliations:** 1 Campus Apucarana, Universidade Tecnológica Federal do Paraná, Apucarana 86812-460, Brazil; renanjacobcarvalho@hotmail.com (R.J.C.); lucasv@alunos.utfpr.edu.br (L.A.V.); 2 Departamento de Informática e Estatística, Universidade Federal de Santa Catarina, Florianópolis 88040-900, Brazil; luiz.nakamura@ufsc.br; 3 Alvaz Agritech, Londrina 86050-268, Brazil; ajrighetto@gmail.com; 4 Instituto de Matemática e Estatística, Universidade de São Paulo, São Paulo 05508-090, Brazil; cpereira@ime.usp.br

**Keywords:** beyond mean regression, distributional regression, parsimony principle, regression models, smoothing functions

## Abstract

This paper presents a discussion regarding regression models, especially those belonging to the location class. Our main motivation is that, with simple distributions having simple interpretations, in some cases, one gets better results than the ones obtained with overly complex distributions. For instance, with the reverse Gumbel (RG) distribution, it is possible to explain response variables by making use of the generalized additive models for location, scale, and shape (GAMLSS) framework, which allows the fitting of several parameters (characteristics) of the probabilistic distributions, like mean, mode, variance, and others. Three real data applications are used to compare several location models against the RG under the GAMLSS framework. The intention is to show that the use of a simple distribution (e.g., RG) based on a more sophisticated regression structure may be preferable than using a more complex location model.

## 1. Introduction

With the increasing use of new data analysis techniques, mainly artificial intelligence, machine learning, neural networks, and big data, regression analysis has become, perhaps, the most important tool among the various statistical (learning) methods of optimization, and of decision-making management. Evidently, the greater the complexity of the databases, the greater the complexity in the proper treatment of these data. The number of papers with increasingly complex techniques is naturally emerging because of the need to extract more accurate information from the data.

This manuscript is more of a work belonging to this class of papers, although we think it is less complex compared to its alternatives, which are mainly presented as (log linear) location models. Usually, the location parameters are associated to other important parameters like mean, percentiles, standard deviation, skewness, and kurtosis, in which these characteristics are implicitly modeled. There are papers that perform a good work obtaining the solutions. For instance: the three parameter log-xgamma Weibull regression model [1], the four parameter Topp Leone generated Burr XII [2], log-odd log-logistic Marshal Olkin generalized half-normal [3] and log-beta Burr XII [4] regression models, and the five parameter log-Hjorth Weibull regression model [5]. We note that many of these complex distributions suffer from the interpretation of the parameters and their estimations needed whenever predictions are demanded.

In the sequel, instead of developing and considering highly complex location models to deal with complex data, a different approach will be used, considering a more sophisticated class of regression models based on the reverse Gumbel (RG) distribution, a simple distribution with simple parameters and predictions interpretations. The chosen tool for the presented analyses is the generalized additive models for location, scale, and shape (GAMLSS) [6] framework, since they allow that any and all of distribution parameters to be explicitly modeled.

Hence, the aim of this paper is to compare if a GAMLSS model based on a very simple distribution (RG) is able to outperform several highly complex location models. In this sense, Section 2 presents a description of the location models, the GAMLSS framework and some statistical inference concepts. In Section 3 we present three real data applications (voltage data, class-H insulation, and heart transplant) comparing some recently developed location models against the RG distribution under the GAMLSS framework. Finally, Section 4 ends the paper with some concluding remarks.

## 2. Materials and Methods

### 2.1. Location Models

Location regression models are useful to relate a dependent (response) variable to one or more explanatory variables. Suppose a response *Y*, with location parameter μ(v), which depends on the explanatory variable vector v. For this case, a class of regression models for location is characterized by

(1) $$\begin{array}{c} Y=\mu (v)+Z, \end{array}$$

where *Z* follows a specific distribution that does not depend on v.

For instance, let us consider that *Y* follows a reverse Gumbel distribution (RG), i.e., *Y*∼RG(μ,σ), also known as the type I extreme value distribution, given by

$$\begin{array}{c} f\left(y;\mu ,\sigma \right)=\frac{1}{\sigma }\exp\left[-\left(\frac{y-\mu }{\sigma }\right)-\exp\left(-\frac{y-\mu }{\sigma }\right)\right],\;-\infty <y<+\infty , \end{array}$$

where −∞<μ<+∞ is the mode, and σ>0 is the scale parameter, E(Y)=μ+0.57722σ and the median is μ+0.36611σ [7]. The RG distribution is appropriate for moderately positive skew data.

Considering that *Z* follows a standard RG distribution, i.e., μ=0, in Equation (1), then *Y* will follow a RG distribution with model parameters θ=(μ(v),σ). Note that, by modeling only μ, we are actually explicitly modeling the mode of the response and also implicitly modeling both the average and median of *Y*.

### 2.2. GAMLSS Framework

An alternative approach, when other measures are affected by explanatory variables, e.g., variance, skewness, and excess of kurtosis, is to explicitly model the parameters related to these measures. In this sense, the GAMLSS framework [6] occupies a prominent position among the beyond the mean (or location) regression models [8], generalizing both generalized linear [9] and generalized additive [10] models. GAMLSS are semi-parametric regression models in which any distribution may be defined to describe the response *Y*, and different regression structures may be considered to explain any or all of its parameters, using linear and/or nonlinear functions.

Let Y∼D(θ), where D is the distribution of the response variable, and θ is its parameter vector. Then, a GAMLSS can be written as

(2) $$\begin{array}{c} g_{k}\left(\theta_{k}\right)=X_{k}\beta_{k}+\sum_{j=1}^{J_{k}}s_{jk}\left(x_{jk}\right), \end{array}$$

where $g_{k}\left(\cdot \right)$ denote appropriate link functions for the *k*th parameter, which is usually determined by the range of the parameter considered [11], $X_{k}$ is a known $n\times (m_{k}+1)$ model matrix, $m_{k}$ denotes the number of explanatory variables related to the *k*th parameter, $\beta_{k}={\left(\beta_{0k},\beta_{1k},\ldots ,\beta_{m_{k}k}\right)}^{⊤}$ is a parameter vector of length $\left(m_{k}+1\right)$, and $s_{jk}\left(.\right)$ are smoothing functions (in this paper, it will be considered as a P-spline [12,13]). When $\sum_{j=1}^{J_{k}}s_{jk}\left(x_{jk}\right)=0$, model (2) reduces to a fully parametric GAMLSS version [6] (pGAMLSS, for short).

Since any distribution may be used in GAMLSS, usually there is no need to transform the data in study, resulting in clearer interpretations. A wide list of distributions in GAMLSS may be found in Reference [7]. For instance, if *Y*∼RG(μ,σ), then a GAMLSS model based on the RG distribution is given by

$$\begin{array}{ccc} & & g_{1}\left(\mu \right)=\mu =X_{1}\beta_{1}+\sum_{j=1}^{J_{1}}s_{j1}\left(x_{j1}\right),\\ & & g_{2}\left(\sigma \right)=\log\sigma =X_{2}\beta_{2}+\sum_{j=1}^{J_{2}}s_{j2}\left(x_{j2}\right). \end{array}$$

Here, the considered link functions for μ and σ were the identity and logarithm due to their range, respectively. Moreover, we can actually rewrite a location model in terms of the GAMLSS framework. Let us consider *Y*∼RG(μ,σ) again, and then Equation (1) can be rewritten as

$$\theta =\left[\begin{array}{c} g_{1}\left(\mu \right)\\ \sigma \end{array}\right]=\left[\begin{array}{c} X_{1}\beta_{1}\\ \sigma \end{array}\right].$$

It is noteworthy that, depending on the parameterization of the response variable distribution [7], μ is not necessarily a location parameter. Nonetheless, the model presented in Equation (2) can be applied more generally to any type of parameter from a population distribution [6].

### 2.3. Estimation and Model Selection

The maximum likelihood estimates for a GAMLSS model can be performed in the gamlss package [14] (and its add-ons) in R software [15]. The algorithms used are the RS and CG procedures described by References [6,11,14] and are available in the documentation of the package.

In order to deal with censored observations (events that will occur in the future) within the GAMLSS framework, the methodology is identical to the one used in classical models, i.e, we must add the probability that this information will occur in the future $1-F(y_{i};\theta_{k})$ into the likelihood, where F(·) denotes the cumulative density function. Then, the log-likelihood is given by $l\left(\theta_{k}\right)=\sum_{i\in F}\log f\left(y_{i};\theta_{k}\right)+\sum_{i\in C}\log1-F\left(y_{i};\theta_{k}\right)$. Computationally, we can use the gamlss.cens [16] package to obtain the model estimates in the presence of censored observations.

As the explanatory variables can be included in any of the regression structures of all parameters, there are some procedures to select the additive terms. In this paper, we are using the so-called Strategy A [11,17], a stepwise-based method applied to select the terms for each model parameters based on the Akaike information criterion (AIC) [18]. This approach can be achieved using the stepGAICAll.A() function in the gamlss package.

After selecting the additive terms, we verify the model assumptions by conducting a residual analysis. The worm plots (WP) [19] are a useful tool based on the normalized quantile residuals [20], that graphically show if the fitted model presents an adequately fit to the data. With this plot we can compare the differences between the empirical and model residual mean, variance, skewness, and kurtosis, respectively, within the range in the QQ plot. More information regarding WP may be found in Reference [11].

## 3. Results

In this section, we will consider three classical data sets that were used as motivational examples to develop new (log-)location models in the past few years. These models will be compared to the GAMLSS framework based on the two-parameter Reverse Gumbel distribution [7]. All comparisons are made using both AIC [18] and Bayesian information criterion (BIC) [21]. We also provide, in each application, the effective degrees of freedom for all fitted models, i.e., the sum of the degrees of freedom of linear terms with the smoothing parts (when they are considered in the fitting process).

### 3.1. Application 1: Voltage Data

This data set was reported by Lawless [22], who conducted an experiment considering accelerated voltage life test to study specimens of solid epoxy electrical-insulation. Basically, the experiment consists in determining the failure times for epoxy insulation specimens (in min), considering three levels of voltage ($x_{i}$): 52.5, 55.0, and 57.5 kV. The total of times observed were n=60, where six observations were classified as censored observations.

These data have already been modeled by the following (log-)location models:

- Five-parameter log-Topp Leone generated Burr XII (LTLGBXII) [2] distribution;
- Four-parameter log-Weibull Marshall-Olkin Weibull (LWMOW) [23] distribution;
- Four-parameter log-Zografos-Balakrishnan odd log-logistic generalized half-normal (LZBOLL-GHN) [24] distribution;
- Four-parameter log-odd log-logistic Fréchet (LOLLFr) [25] distribution;
- Four-parameter heteroscedastic log-extended generalized odd half-Cauchy Weibull (HLEGOHC-W) distribution; four-parameter log-extended generalized odd half-Cauchy Weibull (LEGOHC-W) distribution; two-parameter heteroscedastic log-Weibull (HLW) [26] distribution;
- Three-parameter log-odd log-logistic generalized half-normal (LOLLGHN) distribution; two-parameter log-generalized half-normal (LGHN) distribution; four-parameter log-beta generalized half-normal (LBGHN) [27] distribution;
- Four-parameter log-gamma extended Weibull (LGE-W) [28] distribution
- Four-parameter log-Kumaraswamy generalized Rayleigh (LKwGR); distribution three-parameter log-exponentiated generalized Rayleigh(LEGR) distribution; two-parameter log-generalized Rayleigh (LGR) [29] distribution;
- Four-parameter exponentiated logistic geometric type I(ELGI) distribution; four-parameter exponentiated logistic geometric type II (ElGII) distribution [30].

Note that, as mentioned in Section 2.2, no transformation on the response variable is necessary while using the GAMLSS framework. However, in this application, as considered in the above papers, we will model the logarithm of the failure times, i.e, the response variable considered in this example is *y*: log-time in minutes. Further, $x_{i}$ will be considered as continuous (as in the previous applications), since the goal here is not to check if there is a significant difference between the levels of voltage $x_{i}$ but to understand how *x* impacts in the failure times.

**Figure 1:**
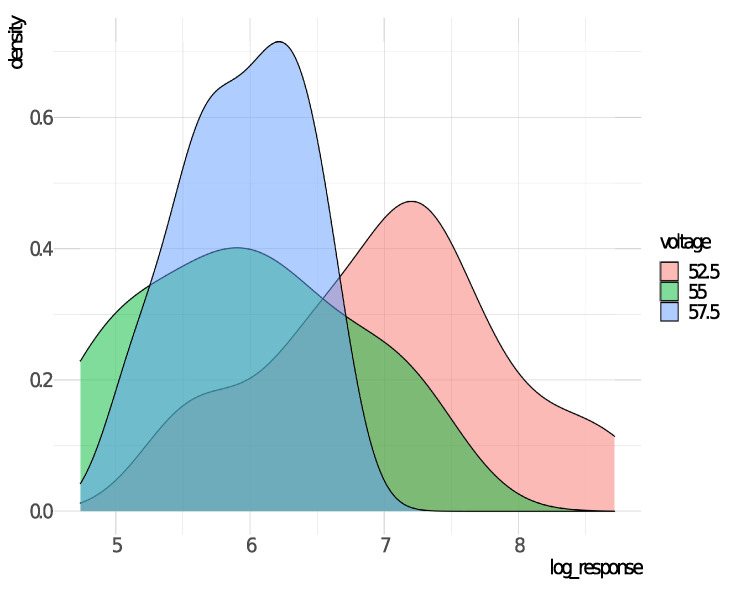
Densities of *y* for each voltage level, disregarding censored observations.

Figure 1 displays the densities of the response variable (log-time in minutes) for each voltage level. The idea here is to check whether it is necessary to fit a regression structure (consider $x_{i}$) for the scale parameter σ of the RG distribution on the GAMLSS framework. As we can see, there is clearly a difference between the dispersion of the three different levels and thus σ may be modeled as a function of $x_{i}$. Moreover, we may note that the mode for $x_{i}=57.5$ and $x_{i}=55.0$ seem to be quite similar, but different from the mode presented by $x_{i}=52.5$, indicating a non linearity effect between μ and the voltage levels.

Based on the Strategy A variable selection method [11,17], the final fitted GAMLSS model, to represent *Y* is given by

(3) $$\begin{array}{c} \mu_{i}=15.646+s\left(x_{i}\right)\;\mathrm{and}\;\log\sigma_{i}=5.83+s\left(x_{i}\right). \end{array}$$

Note that, for both regression structures, a P-spline [12,13] was considered due to the nonlinear relationship between $x_{i}$ and both parameters. The smoothing parameters λ for μ and σ are 3.23 and 2.85, respectively.

In order to show the advantage of the fitted GAMLSS model (3), Table 1 presents all AIC, BIC, and effective degrees of freedom values for all models considered to fit such data. In addition of the semiparametric GAMLSS model presented in (3), we also provide the results of the fully parametric GAMLSS (pGAMLSS) based on the RG distribution, which regression structures are given by $\mu_{i}=13.157-0.129\;x_{i}$ and $\log\sigma_{i}=6.073-0.113\;x_{i}$. The idea here is to show how much reduction in AIC and BIC is caused by the addition of a smoother in the GAMLSS framework (please note that this addition may occur based on practical reasons, i.e., when a nonlinear effect is observed between an explanatory variable and a given parameter). Further, we shall highlight that the maximum likelihood estimates (MLEs), as well as AIC and BIC values presented in Reference [23], seem slightly off for the LWMOW model and the results presented in Table 1 differ from their original paper. The same occurs with the AIC and BIC values for the ELGII model available in Reference [30].

**Figure 2:**
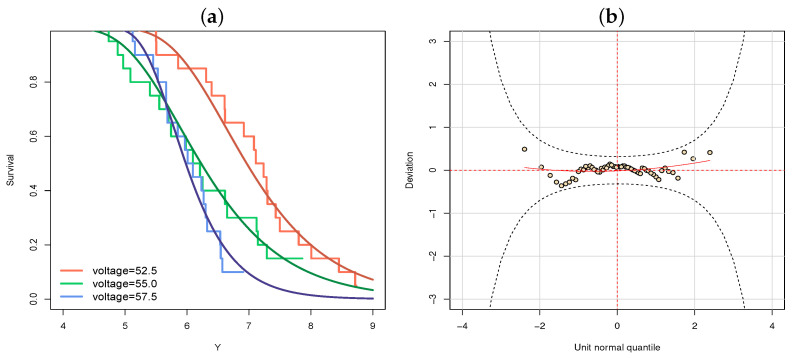
For the voltage data: (**a**) the estimated and empirical survival function from the generalized additive models for location, scale, and shape (GAMLSS) model based on the reverse Gumbel (RG) distribution considering smoothing functions and (**b**) the worm plot (WP).

Table 1 illustrates that the GAMLSS model, based on the RG distribution considering smoothing functions, outperformed all other previous fitted models, i.e., a more flexible class of regression model (GAMLSS) is able to capture more information provided by the data, granting good fit even when a very simple distribution (RG) is considered. Nonetheless, even the parametric GAMLSS version, i.e., the pGAMLSS based on the RG distribution, presents a better fit than all other (log-)location models considered, according to the BIC measure (170.5). Figure 2 displays the fitted survival functions based on the RG distribution and its residuals analysis through the WP. These plots indicate that the proposed model provides a reasonable fit to these data.

### 3.2. Application 2: Class-H

We are now considering the data set about failure of motorettes with a new Class-H insulation. These data were introduced by Nelson [31], where the response variable *y* is the logarithm of the failure time (in hours). In order to investigate the effects of the temperatures in the failure times, four temperatures were considered in this experiment, 190, 220, 240, and 260 ${}^{\circ }$C.

As in previous applications to these data, we will consider the temperature as a continuous variable, i.e., we are not only interested to test the difference between the levels of temperature. Once again, in order to compare previous works with the GAMLSS framework, the RG distribution will be considered. The previous (log-)location models considered to model these data are:

- Four-parameter log-Lomax Weibull (LLW) distribution [32];
- Five-parameter log-beta transmuted Weibull (LBTW) distribution [33];
- Five-parameter log-beta exponentiated Weibull (LBEW) distribution [34];
- Four-parameter log-beta-Weibull (LBW) distribution [35].

**Figure 3:**
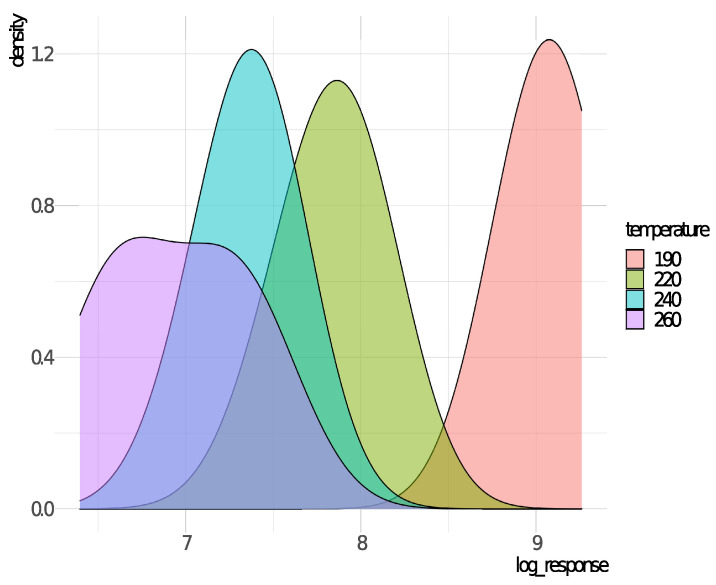
Densities of *y* for each temperature level.

Figure 3 displays the densities for each temperature level. With this plot, we have a visual of information indicating that both parameters, μ and σ, may be modeled by the explanatory variable. We may also note a possible nonlinearity of the temperature effect in mode μ parameter, since the mode for temperature 190 ${}^{\circ }$C is quite lower than the other levels.

Through the Strategy A variable selection method [11,17], the final fitted GAMLSS model based on the RG distribution is given by

$$\begin{array}{c} \mu_{i}=14.966+s\left({\mathrm{temperature}}_{i}\right)\;\mathrm{and}\;\log\sigma_{i}=-4.276+0.011\;{\mathrm{temperature}}_{i}, \end{array}$$

where the fitted smoothing parameter λ for μ is 17.47. Note that, although temperature was considered to model both regression structures, the smoothing function was only necessary to model the mode μ.

Table 2 shows the values of AIC, BIC, and effective degrees of freedom values for all fitted models to the Class-H data. As in the previous application, we also provide the results of the pGAMLSS framework (i.e., only considering linear effects on both parameters) based on the RG distribution, which regression structures are given by $\mu_{i}=15.314-0.033\;{\mathrm{temperature}}_{i}$ and $\log\sigma_{i}=-3.444+0.008\;{\mathrm{temperature}}_{i}$. Once again, we can conclude that, by using a simpler distribution, but considering a flexible regression structure (as GAMLSS), we may have better goodness-of-fit measures.

**Figure 4:**
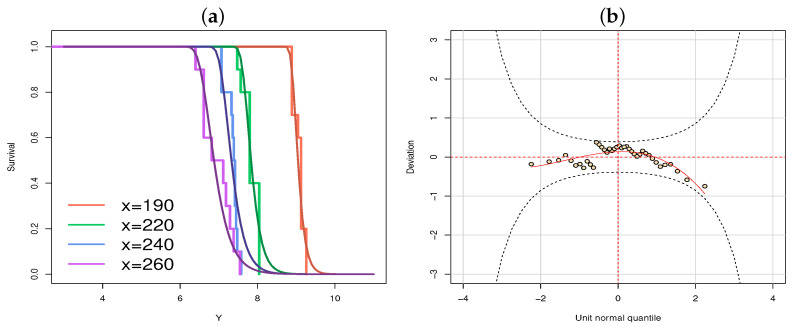
For class-H data: (**a**) the estimated and empirical survival function from RG and (**b**) the WP.

For a visual check of the goodness-of-fit, Figure 4 provides the fitted and empirical survival functions, as well the residuals WP from the fitted GAMLSS model based on the RG distribution, where it seems that the model is adequately fitted to the data.

### 3.3. Application 3: Heart

In this last application, we are considering the data provided by Kalbfleish and Prentice [36], where a study regarding the longevity of patients waiting for a heart transplant was conducted. During the study, some patients (27%) died before an appropriate heart could be found, so, by considering the response variable the time to receive the transplant, these events were considered as censored information.

The goal here is to study the effects of some explanatory variables on the time until transplant. The variables taken into account are *y*: log-time in days since acceptance into the transplantation program to transplant and to death; $\delta_{i}$: failure indicator (0: censored, 1: observed); $x_{i1}$: age at acceptance (in years); $x_{i2}$: previous surgery (0: no, 1: yes); and $x_{i3}$: transplant (0: no, 1: yes).

**Figure 5:**
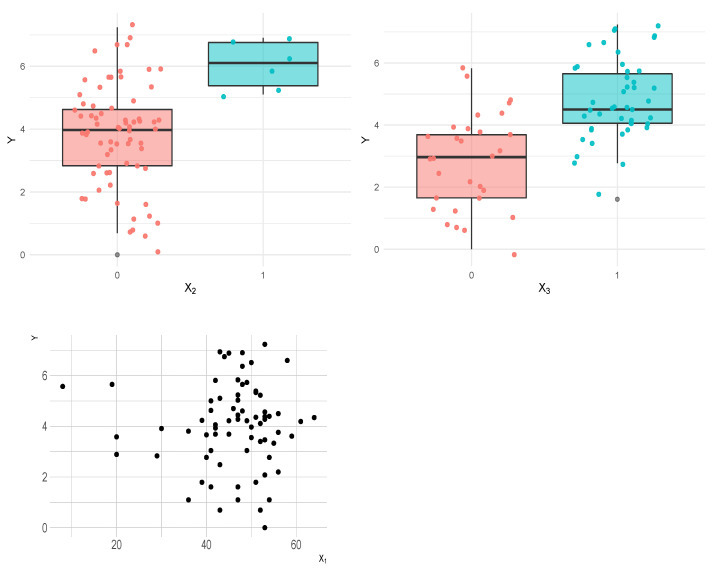
Dispersion plot and boxplots for heart data as a function of the explanatory variables.

Figure 5 shows the relationship between the response and all explanatory variables. We may note that the mode of *y* changes for each level of $X_{2}$ and $X_{3}$, and, as the age at acceptance increases, the mode of *y* decreases, indicating that all three variables might be used to fit the mode μ parameter of the RG distribution. We may also note that the dispersion is influenced by $X_{1}$ and $X_{2}$, indicating that they are probably good predictors to fit the scale parameter σ.

Using the Strategy A variable selection method [11,17], the final fitted GAMLSS model based on the RG distribution is given by

(4) $$\begin{array}{c} \mu_{i}=4.662-0.054x_{i1}+1.768x_{i2}+2.633x_{i3}\;\mathrm{and}\;\log\sigma_{i}=1.967-0.033x_{i1}. \end{array}$$

No smoothing functions were applied onto the age at acceptance in both parameters, i.e., in fact, the final selected GAMLSS model to explain the behavior of the response variable according to the available explanatory variables is the fully parametric version, pGAMLSS. As stated in the first application in Section 3.1, the smoothing functions may be considered when there is a nonlinear effect of a explanatory variable in a given parameter (which is not observed in this case).

We will compare model (4) with the following (log-)location models already proposed in the literature to deal with these data:

- Four-parameter log-odd power Lindley Weibull (LOPLW) distribution [37];
- Four-parameter log-extended odd Fréchet generalized half-normal (LEOF-GHN) distribution [38];
- Four-parameter log-extended-exponentioned Weibull (LEE-W) distribution [39];
- Four-parameter log-Burr XII-Weibull (LBXII-W) distribution [40];
- Four-parameter log-log-gamma generated-Weibull (LLGG-W) distribution [41];
- Four-parameter log-Topp-Leone odd log-logistic-Weibull (LTLOLL-W) distribution [42];
- Three-parameter log-odd log-logistic Weibull (LOLLW) distribution [43].

**Figure 6:**
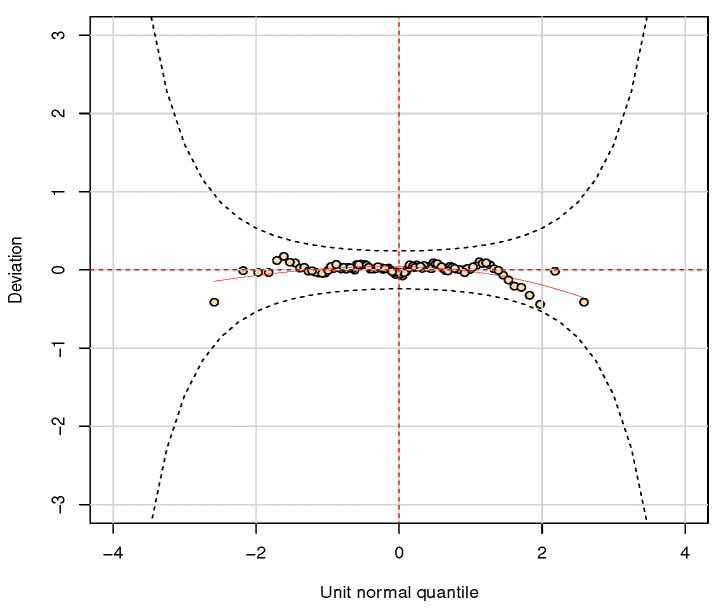
Worm plot of the fitted fully parametrc GAMLSS model based on the RG distribution.

Table 3 presents the AIC, BIC, and effective degrees of freedom values. Even though the LEOF-GHN model presents the smallest AIC, the pGAMLSS based on the RG distribution returns an AIC of only 1.5 units greater. Moreover, the RG model based on the fully parametric GAMLSS framework produces the best BIC value; thus, by the parsimony principle and also considering the model with the simplest interpretability, the GAMLSS alternative would be preferable. In order to check the model assumptions, the WP of the fitted pGAMLSS model is presented in Figure 6, showing that, in fact, the model provides a reasonable fit. Since there is a continuous covariate in this problem, we do not present the estimated and empirical survival functions.

## 4. Discussion

Although there is a reasonable number of new regression models being developed in the last few years (e.g., the ones previously fitted to the three applications considered in this paper), usually they present a highly complex structure that may suffer from the interpretation of the parameters. This is a critical drawback since the interpretability of such characteristics is still the major advantage of regression models compared to other methods.

The key point within the discussion in application sections in papers that develop new (log-)location models is usually based on goodness-of-fit measures, such as AIC and BIC. Focusing on this specifically point, let us suppose a response variable *Y* that follows a Gaussian distribution, and an explanatory variable *X* which directly affects both the mean μ and standard deviation σ of *Y*. To fit such behaviors, should we build a location model or a heteroscedastic model (GAMLSS in other words) or propose a new location model? The natural choice here seems to be the GAMLSS (distributional regression) approach.

Further, we may be interested in discussion on why more complex models might present better statistics, like AIC and BIC, when compared to some of their special and/or limiting cases. Looking at the properties of these models, we usually note the association between their location parameter and other important characteristics, such as mean, percentiles, standard deviation, skewness, and kurtosis. This means that, in the modeling stage of the location parameter, we are implicitly modeling these characteristics, as well. In the GAMLSS structure, we can explicitly model any and all parameters directly, i.e., different regression structures can be considered to explain all the parameters of the response variable distribution. Thus, apart from producing better goodness-of-fit measures, we can still identify which characteristics affect each of the parameters.

Finally, we present a review of regression models, based on fitting any and all parameters using linear and/or nonlinear structures, and consequently modeling more accurately the data behavior through the GAMLSS framework. The use of simpler models, with interpretable parameters, based on very sophisticated regression structures, presented better results than the ones obtained through highly complex location models. Following the parsimony principle and/or the interpretability of the parameters, we may conclude—at least from a practical point of view—that, by using the GAMLSS framework, the development and proposal of new models with a high number of parameters is, in some cases, avoidable.

## Figures and Tables

**Table 1:** Akaike information criterion (AIC), Bayesian information criterion (BIC), and effective degree of freedom (df) from the fitted models for the voltage data.

Model	df	AIC	BIC	Model	df	AIC	BIC
RG (GAMLSS)	5.5	157.6	168.6	LGE-W	5	168.6	179.1
HLEGOHC-W	6	161.3	173.9	HLW	4	171.4	179.8
RG (pGAMLSS)	4	162.1	170.5	log-Weibull	3	173.4	179.7
LOLLFr	4	164.3	172.7	LWMOW	5	173.5	184.0
LEGOHC-W	5	165.6	176.1	LKwGR	5	177.4	187.8
LZBOLL-GHN	5	166.2	176.7	LGHN	3	178.8	185.1
LOLLGHN	4	166.4	174.8	LGR	3	179.5	185.7
ELGI	5	166.7	177.2	LEGR	4	180.5	188.8
LBGHN	5	167.1	177.5	ELGII	5	187.3	197.8
LTLGBXII	6	168.4	180.9				

**Table 2:** AIC, BIC, and effective degree of freedom (df) from the fitted models for the Class-H data.

Model	df	AIC	BIC
RG (GAMLSS)	5.3	4.29	11.0
LLW	5	13.8	22.2
RG (pGAMLSS)	4	16.6	23.3
LBEW	6	16.9	27.0
LBTW	6	18.5	28.6
LBW	5	18.7	27.2
log-Weibull	3	22.4	27.5

**Table 3:** AIC, BIC, and effective degree of freedom (df) from the fitted models for the heart data.

Model	df	AIC	BIC
LEOF-GHN	8	334.3	355.3
RG (pGAMLSS)	7	335.8	354.2
LOPLW	8	338.4	359.5
LEE-W	7	343.3	361.8
LBXII-W	7	343.3	361.8
LTLOLL-W	8	345.3	366.4
LLGG-W	7	345.7	364.1
LOLLW	6	347.5	363.4
log-Weibull	5	353.4	366.6

## Data Availability

The data presented in this study are available in Lawless [22], Nelson [31], and Kalbfleish and Prentice [36]. Further, all codes used in this paper are available at https://colab.research.google.com/drive/1Hlyb6nsqJ3aLiqc8kYRMYYduYfWwMZ0O?usp=sharing.

## References

[1] Cordeiro G.M., Altun E., Korkmaz M.Ç., Pescim R.R., Afify A.Z., Yousof H.M. (2020). The xgamma Family: Censored Regression Modelling and Applications. Revstat. Stat. J..

[2] Yousof H.M., Altun E., Rasekhi M., Alizadeh M., Hamedani G.G., Ali M.M. (2019). A new lifetime model with regression models, characterizations and applications. Commun. Stat. Simul. C.

[3] Korkmaz M.C., Altun E., Alizadeh M., Yousof H.M. (2019). A new flexible lifetime model with log-location regression modeling, properties and applications. J. Stat. Manag. Syst..

[4] Afify A.Z., Cordeiro G.M., Bourguignon M., Ortega E.M.M. (2018). Properties of the transmuted Burr XII distribution, regression and its applications. J. Data Sci..

[5] Korkmaz M.Ç., Altun E., Yousof H.M., Hamedani G.G. (2020). The Hjorth’s IDB generator of distributions: Properties, characterizations, regression modeling and applications. J. Stat. Theory Appl..

[6] Rigby R.A., Stasinopoulos D.M. (2005). Generalized additive models for location, scale and shape. J. R. Stat. Soc. Ser. C (Appl. Stat.).

[7] Rigby R.A., Stasinopoulos D.M., Heller G.Z., De Bastiani F. (2019). Distributions for Modeling Location, Scale and Shape: Using GAMLSS in R.

[8] Kneib T. (2013). Beyond mean regression. Stat. Model..

[9] Nelder J.A., Wedderburn R.W.M. (1972). Generalized linear models. J. R. Stat. Soc. Ser. A (Gen.).

[10] Hastie T.J., Tibshirani R.J. (1990). Generalized Additive Models.

[11] Stasinopoulos D.M., Rigby R.A., Heller G.Z., Voudouris V., De Bastiani F. (2017). Flexible Regression and Smoothing: Using GAMLSS in R.

[12] Eilers P.H., Marx B.D. (1996). Flexible smoothing with B-splines and penalties. Stat. Sci..

[13] Eilers P.H.C., Marx B.D., Durbán M. (2015). Twenty years of P-splines. SORT.

[14] Stasinopoulos D.M., Rigby R.A. (2007). Generalized additive models for location scale and shape (GAMLSS) in R. J. Stat. Softw..

[15] R Core Team (2020). R: A Language and Environment for Statistical Computing.

[16] Stasinopoulos M., Rigby B., Mortan N. (2018). gamlss.cens: Fitting an Interval Response Variable Using ‘gamlss.family’ Distributions.

[17] Ramires T.G., Nakamura L.R., Righetto A.J., Pescim R.R., Mazucheli J., Rigby R.A., Stasinopoulos D.M. (2021). Validation of stepwise-based procedure in GAMLSS. J. Data Sci..

[18] Akaike H. (1974). A new look at the statistical model identification. IEEE Trans. Autom. Control.

[19] van Buuren S., Fredriks M. (2001). Worm plot: A simple diagnostic device for modelling growth reference curves. Stat. Med..

[20] Dunn P.K., Smyth G.K. (1996). Randomized quantile residuals. J. Comput. Graph Stat..

[21] Schwarz G. (1978). Estimating the dimension of a model. Ann. Stat..

[22] Lawless J.F. (2003). Statistical Models and Methods for Lifetime Data.

[23] Korkmaz M.Ç., Cordeiro G.M., Yousof H.M., Pescim R.R., Afify A.Z., Nadarajah S. (2019). The Weibull Marshall-Olkin family: Regression model and application to censored data. Commun. Stat. Theory Methods.

[24] Altun E., Yousof H.M., Hamedani G.G. (2018). A new generalization of generalized half-normal distribution: Properties and regression models. J. Stat. Dist. Appl..

[25] Yousof H.M., Altun E., Hamedani G.G. (2018). A new extension of Fréchet distribution with regression models, residual analysis and characterizations. J. Data Sci..

[26] Cordeiro G.M., Ramires T.G., Ortega E.M.M., Alizadeh M. (2017). The new family of distributions and applications in heteroscedastic regression analysis. J. Stat. Theory Appl..

[27] Pescim R.R., Ortega E.M., Cordeiro G.M., Alizadeh M. (2017). A new log-location regression model: Estimation, influence diagnostics and residual analysis. J. Appl. Stat..

[28] Cordeiro G.M., Nadarajah S., Ortega E.M., Ramires T.G. (2016). An alternative two-parameter gamma generated family of distributions: Properties and applications. Hacet. J. Math. Stat..

[29] Gomes A.E., da-Silva C.Q., Cordeiro G.M., Ortega E.M.M. (2014). A new lifetime model: The Kumaraswamy generalized Rayleigh distribution. J. Stat. Comput. Simul..

[30] Mendoza N.V.R., Ortega E.M.M., Cordeiro G.M. (2016). The exponentiated-log-logistic geometric distribution: Dual activation. Commun. Stat. Theory Methods.

[31] Nelson W.B. (2009). Accelerated Testing: Statistical Models, Test Plans, and Data Analysis.

[32] Cordeiro G.M., Ortega E.M.M., Popović B.V., Pescim R.R. (2014). The Lomax generator of distributions: Properties, minification process and regression model. Appl. Math. Comput..

[33] Pal M., Tiensuwan M. (2014). The beta transmuted Weibull distribution. Austrian J. Stat..

[34] Cordeiro G.M., Gomes A.E., da-Silva C.Q., Ortega E.M.M. (2013). The beta exponentiated Weibull distribution. J. Stat. Comput. Simul..

[35] Lee C., Famoye F., Olumolade O. (2007). Beta-Weibull distribution: Some properties and applications to censored data. J. Mod. Appl. Stat. Methods.

[36] Kalbfleisch J.D., Prentice R.L. (1980). The Statistical Analysis of Failure Time Data.

[37] Korkmaz M.C., Altun E., Yousof H.M., Hamedani G.G. (2019). The odd power Lindley generator of probability distributions: Properties, characterizations and regression modeling. Int. J. Stat. Probab..

[38] Yousof H.M., Rasekhi M., Altun E., Alizadeh M. (2019). The extended odd Fréchet family of distributions: Properties, applications and regression modeling. Int. J. Math. Comput..

[39] Alizadeh M., Afshari M., Hosseini B., Ramires T.G. (2018). Extended exp-G family of distributions: Properties, applications and simulation. Commun. Stat. Simul. Comput..

[40] Cordeiro G.M., Yousof H.M., Ramires T.G., Ortega E.M.M. (2018). The Burr XII system of densities: Properties, regression model and applications. J. Stat. Comput. Simul..

[41] Cordeiro G.M., Bourguignon M., Ortega E.M.M., Ramires T.G. (2018). General mathematical properties, regression and applications of the log-gamma-generated family. Commun. Stat. Theory Methods.

[42] Brito E., Cordeiro G.M., Yousof H.M., Alizadeh M., Silva G.O. (2017). The Topp-Leone odd log-logistic family of distributions. J. Stat. Comput. Simul..

[43] Cruz J.N.D., Ortega E.M.M., Cordeiro G.M. (2016). The log-odd log-logistic Weibull regression model: Modelling, estimation, influence diagnostics and residual analysis. J. Stat. Comput. Simul..

